# Effects of a native avian predator (weka; *Gallirallus australis*) and colony density on breeding success of a burrow-nesting seabird (tītī; *Ardenna grisea*)

**DOI:** 10.1371/journal.pone.0346357

**Published:** 2026-04-02

**Authors:** Joanna K. Carpenter, David Fletcher, James Arbuckle, Morgan Coleman, Becky Goodsell, Andrew Gormley, Aurora Metzger, Trent Moss, Kate H. Orwin, Rakiura Tītī Islands Administering Body, Rakiura Tītī Committee, Darren Scott, Luke Sutton, Theo D. W. Thompson, Puke Timoti, Amy Whitehead, Phil O’B. Lyver

**Affiliations:** 1 Bioeconomy Science Institute, Dunedin, New Zealand; 2 David Fletcher Consulting Limited, Karitane, New Zealand; 3 Bioeconomy Science Institute, Lincoln, New Zealand; 4 Invercargill, New Zealand; 5 Tohu Environmental, Invercargill, New Zealand; 6 Department of Conservation, Invercargill, New Zealand; 7 Bioeconomy Science Institute, Hamilton, New Zealand; Hawaii Pacific University, UNITED STATES OF AMERICA

## Abstract

As island ecosystems are restored, native predators are recovering and re-establishing interactions with their prey. However, compared with widely-publicised impacts of invasive predators, little is known about the dynamics and stability of native predator-prey dynamics on islands. Prey species such as seabirds may still be vulnerable to population decline, as even native predation can become unsustainable at a population-level when combined with other threats. Large seabird colonies may buffer against native predation by diluting its per-capita impact, but this will depend on the functional response of native predators to prey densities. Here, we test the impact of a predatory native rail (weka, *Gallirallus australis*) on breeding success of a burrow-nesting seabird (tītī/muttonbird/sooty shearwater, *Ardenna grisea*) that nests in dense colonies on the Rakiura Tītī Islands, southern New Zealand. We used burrowscopes and trail cameras to assess tītī breeding success (*N* = 126 nests) at three sites with weka, and two sites without. We also tested how tītī burrow density (a proxy for colony density) influenced breeding success. Tītī breeding success (probability of an egg hatching and then fledging) varied across sites, ranging from 0.39 to 0.88. Model predictions indicated that weka had a negligible effect on tītī breeding success overall, with breeding success increasing with increasing tītī burrow density, although both relationships had considerable uncertainty. Trail camera data showed weka activity was primarily diurnal, and that weka activity at monitored burrows did not increase during the period nests are most vulnerable (mid-January until late-February). We suggest that weka predation on tītī may be inversely density dependent, and that large colonies absorb the impact of predation. However, if tītī populations decline in the future due to other factors (e.g., climate change, harvest), the impact of weka on breeding success may increase, resulting in accelerated, localized tītī population declines*.*

## Introduction

Predator-prey dynamics are key structuring processes in continental ecosystems [1], yet remain relatively understudied in island systems [but see 2, 3, 4]. Insular faunas are often described as ‘disharmonic’, with simplified communities and limited predation pressure [5]. However, native predators such as raptors or large reptiles are integral to many island ecosystems, and can exert substantial effects on prey populations [6,7]. The arrival of invasive mammal predators has often suppressed or wiped out native predators, eroding their ecological roles [e.g., 7]. However, the restoration of islands through invasive mammal eradication is allowing the recovery of native predators and the reactivation of historically suppressed predator-prey interactions [e.g., 8]. Although these dynamics have generally co-evolved over long time spans, the additional anthropogenic pressures faced by island species sometimes sparks concern that native predation could contribute to prey declines [9,10]. Given that native predators are often threatened and ecologically important, their impacts require careful assessment to inform balanced conservation management.

Seabirds are often an important resource for native predators on islands [11–13], but are also the most threatened group of birds globally due to threats both on land and at sea [14]. Key factors that determine the impact of native predators on seabirds include the abundance and density of the seabird population, and the functional response of predators to those densities (i.e. Holling’s type I, II, and III functional responses). Many seabird species nest in extremely dense aggregations, which may mitigate the effects of predators [15,16] through facilitating communal defence against predators (e.g., mobbing) or by diluting the effect of predation and effectively swamping predators [17,18]. Swamping effects could occur if predators show a functional response in which their predation plateaus at high prey densities (i.e., a Holling’s type II or type III functional response, cf. a type I functional response where predation has a more linear relationship with prey density). However, many seabird breeding colonies have greatly reduced densities due to anthropogenic pressures, and this could impair their ability to absorb the effect of native predators [19,20]. For example, culturally important harvests of seabird species, or mortality driven by fisheries’ bycatch, could result in lower colony densities that then exacerbate the effect of predation by native predators [21]. Conversely, if predators show learned predatory behaviours only above certain prey density thresholds (Holling’s type III functional response), or focus on rich food patches (i.e., high-density prey colonies), then low-density populations may suffer less predation than higher density populations [e.g., 22].

New Zealand (hereafter NZ) is considered the ‘seabird capital of the world’, with approximately 25% of the world’s seabird species breeding there [23]. Historically, burrow-nesting seabirds thrived across NZ, with massive breeding colonies both on the main islands (North and South Islands, Stewart Island/Rakiura [hereafter Rakiura]) and smaller satellite islands [24]. On the main islands, they co-existed with native ground-dwelling predators including reptiles (tuatara, *Sphenodon punctatus*), rails (e.g., weka, *Gallirallus australis*) and adzebills (*Aptornis* spp.), as well as aerial avian predators like the New Zealand falcon / kārearea (*Falco novaeseelandiae*) [7]. Seabird colonies would have been extremely dense in some cases, and this may have buffered populations from predation by swamping native predators [25].

The introduction of invasive predatory mammals to NZ (predominantly rats [*Rattus* spp.], mustelids [*Mustela* spp.], and feral cats [*Felis catus*]) wiped out the majority of these colonies on the main islands, and most colonies now only occur on smaller island refugia free of mammalian predators [23,26]. The Rakiura Tītī Islands (a collection of 35 islands off the coast of Rakiura) are a major refuge for burrow-nesting seabird colonies and collectively harbour an estimated 14.6 million tītī (*Ardenna grisea*), as well as many other smaller burrow-nesting seabirds [27]. Tītī (also called muttonbirds or sooty shearwaters) weigh 650–950 g, and are an abundant, colonial, trans-equatorial migrant seabird. Rakiura Māori (the indigenous people of this region) maintain a customary harvest of tītī chicks on these islands, where a proportion of chicks are harvested in April and May (colloquially termed ‘muttonbirding’). Ship rats (*Rattus rattus*) and kiore (*Rattus exulans*) were present on some of the islands, but were eradicated from most islands in the late 1990s and early 2000s. However, weka (omnivorous, flightless rails weighing 430–1400 g) were introduced to several Tītī Islands from Rakiura over a century ago, and are still present [28]. Although weka are not technically native to these islands, tītī and weka co-evolved together on the main islands of NZ, and the high mobility of tītī limits evolutionary isolation of nearby island populations. The Rakiura Tītī Islands therefore offer a rare opportunity to understand the dynamics between NZ’s native predators and seabirds in the absence of predatory mammals.

Although weka are an important part of NZ ecosystems, and some subspecies are threatened, their presence on offshore islands is often controversial due to their potential predatory impacts on other threatened species [29,30]. A study carried out on Taukihepa/Big South Cape Island (the largest Rakiura Tītī island – hereafter Taukihepa) in the early 2000s found that weka were having an impact on tītī breeding success through predation of eggs and chicks, with an estimated 9.9% of nest failures attributed to weka predation [31]. However, ship rats were still present on the island at that time, and were causing extremely low breeding success for weka [32]. Since ship rats were eradicated on Taukihepa in 2006, weka have increased significantly in abundance [33], but recent impacts of weka on tītī breeding success remain unknown.

Here, we investigated the influence of weka presence and tītī burrow density on tītī breeding success (the probability that an egg hatches and becomes a fledged chick) at mammal-free sites on Rakiura Tītī Islands with and without weka. We also used trail cameras to assess weka activity at the sites with weka to see whether daily weka activity increased during the period when nests are most vulnerable (i.e., mid-January until late-February when chicks are young and often unattended by an adult [31,34]). We hypothesised: 1) that areas of high tītī burrow density would have greater breeding success, reflecting higher-quality nesting habitat, regardless of weka presence; and 2) at sites where weka were present, high-density nesting areas would confer an additional benefit through predator dilution or buffering effects, leading to a more positive relationship between burrow density and breeding success. These paired hypotheses allow for both habitat quality and predator pressure to interact and influence reproductive outcomes. Although negative density-dependent effects have been shown to affect breeding success in some systems [e.g., 35, 36], this was unlikely to be occurring at our sites because burrow densities were too low.

## Methods

### Study sites

**Fig 1:**
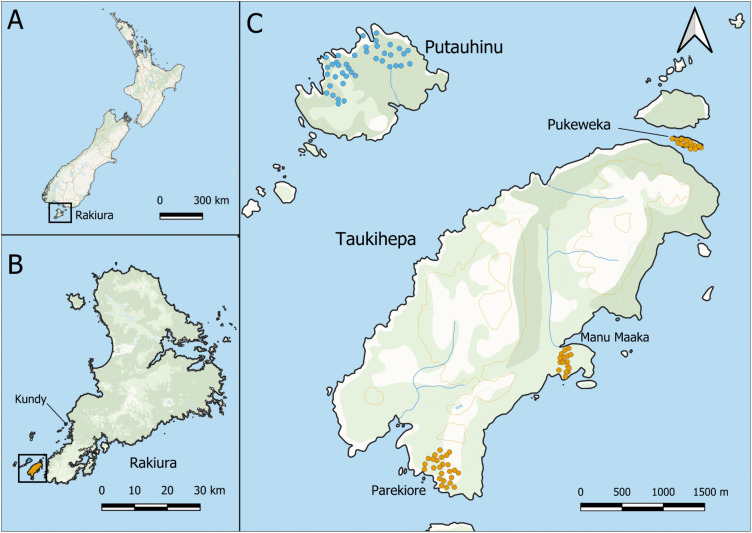
Maps showing study sites and locations. **(A)** Location of the study region within NZ. **(B)** Study islands (shaded orange for weka islands; blue for non-weka islands) in relation to Stewart Island/Rakiura. **(C)** Individual monitored tītī (*Ardenna grisea*) burrows across study sites. Orange dots denote monitored burrows at sites with weka present; blue dots are monitored burrows at sites with weka absent. Note: Panel C only shows four sites but the fifth (Kundy: weka absent) is visible in panel **B.** Maps were created using QGIS 3.44 and are based on NZ Topolite Basemap obtained from Toitū Te Whenua Land Information New Zealand under a CC-BY 4.0 license.

We measured tītī breeding success at five sites across four islands; two islands with weka (Taukihepa [797 ha], and Pukeweka [3 ha]), and two islands without weka (Putauhinu [149 ha] and Kundy [21 ha]; Fig 1). We used two separate sites (Parekiore and Manu Maaka) on Taukihepa due to its large size. Sites were chosen in part based on the willingness of the muttonbirding whānau (families) to support the research, but were also matched as closely as possible for other environmental variables. All five sites have a similar geology (batholithic granite overlaid by a thick layer of peat) and plant communities, although Taukihepa and Putauhinu have more diverse vegetation than the two smaller islands [37]. Monitored tītī nests across all sites were predominantly under canopies of *Macrolearia spp.*, interspersed with occasional *Veronica elliptica, Myrsine chathamica,* and *Dracophyllym longifolium.* Tītī have formed dense colonies across these sites for hundreds of years [38], engineering the islands’ ecosystems through the input of marine nutrients and bioturbation of the peat soils. Burrows are ubiquitous in our monitored areas but the highest density of tītī burrows occurs close to the coastline under forest dominated by *Macrolearia* spp. [39]. The climate is wet, with over 250 rain days (> 0.1 mm per day) throughout the year.

Weka were introduced to Taukihepa sometime between 1913 and 1923 [28], and probably arrived on Pukeweka around the same time (as weka are commonly recorded swimming between the two islands). Weka were also introduced to Kundy but were eradicated in 1984. Under Schedule 3, Part 1 of the New Zealand Wildlife Act 1953 weka can be legally harvested from the Rakiura Tītī Islands by Rakiura Māori, and some hunting of weka happens at Manu Maaka and Pukeweka during the period people are on the islands (mid-March until end of May), but not at Parekiore. All islands are free of introduced small mammalian predators that are known to prey on ground-nesting birds elsewhere in New Zealand: Taukihepa and Pukeweka underwent ship rat eradications in 2006, Putauhinu underwent kiore eradication in 1998, and Kundy has always been free of invasive mammals. Given the length of time since the rodent eradications (minimum 20 years), we expected that tītī and weka would no longer be responding to rat removal. Subantarctic skua / hākoakoa (*Stercorarius antarcticus*) and banded rail / mioweka (*Hypotaenidia philippensis*; a native rail) are the only potential predators other than weka. Mioweka only occur on Kundy, while skua can be present at all sites.

Tītī arrive on the islands in September. Breeding females lay a single egg in late November/early December, which typically hatches in mid–late January. A parent remains with the chick for approximately one week (the ‘guard’ stage) following hatching and then the chick is left alone while the parents take provisioning trips (the ‘post-guard’ stage). Adult tītī from our study islands almost certainly forage in the same areas during the breeding season as other studies have demonstrated that even breeding tītī from highly disparate NZ colonies (e.g., 850 km apart) have overlapping foraging ranges [40,41]. Adult tītī begin to leave the islands to migrate to the Northern Hemisphere in late March, and chicks fledge in late April or May. While all of our study sites are subject to tītī harvests by muttonbirding whānau, people do not arrive on the islands until mid-March, and most harvest at our study sites occurs when chicks have left their burrows from mid-April onwards (i.e., after our study period). Therefore, harvest had no effect on our results.

### Breeding success

We monitored 126 occupied burrows in total (Parekiore *n* = 25; Manu Maaka *n* = 18; Pukeweka *n* = 15; Putauhinu *n* = 40; Kundy *n* = 28) using burrowscopes and trail cameras. Initial monitoring occurred between 30 Nov–6 Dec 2024 (visit 1), when most tītī had just laid eggs. At each site, 15–40 randomly placed points were established, depending on the site size. We aimed to distance points within each site by at least 40 m. At each point, we created a circular plot (3 m radius) and burrowscoped the burrows within it until we found one with a single-entrance burrow occupied by an incubating adult; this became our monitored burrow for that particular point (each point had one monitored burrow). We selected single-entrance burrows to improve our chances of detecting a predation event on camera (as weka could only use one entrance), although we note this could have potentially influenced our results if single-entrance burrows experience different breeding success to multi-entrance burrows. Each monitored burrow was monitored until mid-April using a trail camera (initially Bushnell Core or Reconyx Hyperfire; all Bushnells were replaced mid-season with Reconyx) positioned ~1 m from the entrance. Cameras were motion-triggered with 3-image bursts and no stand-down period. Trail camera images were used to determine causes of nest failure (for failed nests only) and to index weka activity (at weka sites only). For each plot, we also measured tītī burrow entrance density (number of entrances per plot; hereafter burrow density), but not occupancy, and the distance between the entrance and the occupied nest chamber (burrow length) for the monitored nest. Tītī burrow density is often estimated using 3 m radius plots when colonies are too large to be censused [e.g., 27, 42], and burrow densities in these small plots are closely related to burrow densities at larger spatial scales (unpub. data).

Monitored burrows were re-surveyed twice using a burrowscope to assess whether nests were still active: 27–30 January 2025 (visit 2 – post-hatching) and 14–16 April 2025 (visit 3 – c. 1 week before chicks begin to leave their burrows). This last check occurred 2 weeks into the period when Rakiura Māori begin to harvest chicks, but chicks were not harvested from the monitored burrows. Although our last check happened slightly before chicks leave the burrow, we considered a chick present at the last check as a ‘successful nest’ as the chicks are too large at that stage to succumb to weka predation [34]. However, this may overestimate true breeding success because chicks could still die due to starvation or other causes prior to fledging. All potential disturbance and animal manipulations were approved by the Manaaki Whenua – Landcare Research Animal Ethics Committee (Application number 23/01/02).

Trail camera images were reviewed and tagged in ‘Digikam’, an Open Source software package that allows manual data tagging and extraction from trail camera imagery (https://www.digikam.org/). All weka image detections (from weka sites only) were tagged and filtered to ≥ 5-min intervals. Within each 5 min interval, we retained the highest count of weka detected in one image (e.g., if four weka were detected in a photo then a count of ‘4’ was retained for that 5 min interval).

Although we intended cameras to be active over the whole monitoring period, the batteries in many cameras failed (21 of 58 cameras at weka sites) at least 2 weeks before our visit in late January. Our cameras performed better from late January 2025 until our final check in mid-April, but several (*n* = 15) failed during that period too. Therefore, it is likely cameras would have missed predation events, especially in the period shortly after eggs hatched in mid-January. For our assessment of weka activity among sites, we only used trail cameras that were active for at least 80% of the entire monitoring period (*n* = 36 cameras across the three sites). For our assessment of the circadian activity of weka, we only used camera data from the 30 January until the 14 April 2025, and from cameras that were active for at least 80% of this period (*n* = 43 cameras across the three sites).

### Analysis

We assessed the effect of weka presence and burrow density on breeding success using Bayesian logistic regression models, with each regression coefficient in each model having a Normal prior with mean = 0 and variance = 1000. We use the terms ‘stage 1’ (mostly egg stage, but including some early chicks) and ‘stage 2’ (chick stage) to refer to the period between visits 1 and 2, and the period between visits 2 and 3, respectively. For every nest, we were able to detect whether it was active at each stage, and we never had nests which we classified as unsuccessful that were classified as active at a subsequent nest check. This enabled logistic regression to estimate the probability of success or fail at each stage, with three predictors: site, burrow density in a 3 m radius around the nest, and stage (1 or 2). We used WAIC-based (Widely Applicable Information Criterion) model weights to determine the importance of each predictor for prediction, and 95% credible intervals (CIs) to assess the uncertainty of an estimate.

Of the five sites, three were located on islands with weka. Although four site comparisons are required in the model, our primary interest was the contrast between sites with and without weka, hereafter referred to as the “weka” predictor. The regression coefficient for this predictor estimates the difference in breeding success between sites where weka are present and those where they are absent. The remaining three site predictors were included solely to account for residual differences in breeding success among individual sites. These coefficients estimate the following pairwise comparisons: (1) Kundy versus Putauhinu; (2) Pukeweka plus Manu Maaka versus Parekiore; and (3) Pukeweka versus Manu Maaka.

We initially checked whether ‘stage’ was needed in the model (i.e., whether nest failure varied by stage) by fitting a model with all main effects and interactions involving the four predictors for site, plus burrow density and stage, and then dropping stage from this model. We used WAIC-based model weights [43] to compare these two initial models. The model without stage effects was best, with a model weight of 1, so we assumed no stage effect on breeding success. We then fitted all possible models based on the four predictors for site, plus burrow density (and interactions between them), and calculated the corresponding WAIC-based model weights. We then used model averaging based on these weights [43] to provide an estimate and 95% CIs for the probability of nest success, throughout the range of predicted burrow densities, separately for the sites on islands with and without weka (and for the difference between these two types of site).

We also fitted a Bayesian Normal model to assess whether burrows were longer at sites with weka (reflecting learned behaviour by tītī to avoid weka predation). We fitted the model to the logarithm of burrow length, with weka presence as a fixed effect, and site as a Normal random effect. The intercept and weka effect were both given a Normal prior with mean = 0 and variance = 1000, while the prior for both the between-site standard deviation and the error standard deviation was Uniform between 0 and 10.

We used the summed counts of weka detected by each trail camera each day (after removing detections within five minutes of another weka detection) as an index of weka activity, to investigate whether weka activity differed among sites and throughout the monitoring period. We fitted a Bayesian generalised additive model (GAM) to the square-root of the mean daily weka detection for each site and day, as use of a square-root transformation led to the assumptions of a Normal GAM being better satisfied. Site-specific smooth functions of day were estimated using cubic regression splines, where smoothness was controlled by a site-specific penalty parameter (lambda). The spline coefficients for each site were given a multivariate Normal prior with mean zero and precision matrix equal to the product of lambda and the spline penalty matrix. Each lambda was given a Gamma(1, 0.005) prior, the intercept for each site was given a Normal prior with mean = 0 and variance = 1000, and the error standard deviation was given a Uniform prior between 0 and 10.

We fitted all models using the software JAGS [44], implemented using the package rjags in R Version 4.5.2 [45].

## Results

**Fig 2:**
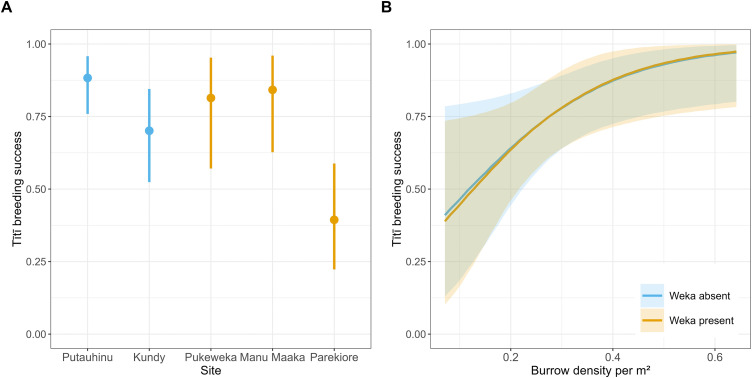
Tītī (*Ardenna grisea*) observed and predicted breeding success across the five study sites on the Rakiura Tītī Islands, NZ. **(A)** Breeding success with 95% credible intervals. Orange bars are sites with weka (*Gallirallus australis*), blue bars are sites without weka. **(B)** Predicted tītī breeding success with and without weka, across the range of observed tītī burrow densities that are shared by all five sites. Shading shows 95% credible intervals. All predictions derived from model averaging across the models shown in Table 1.

**Fig 3:**
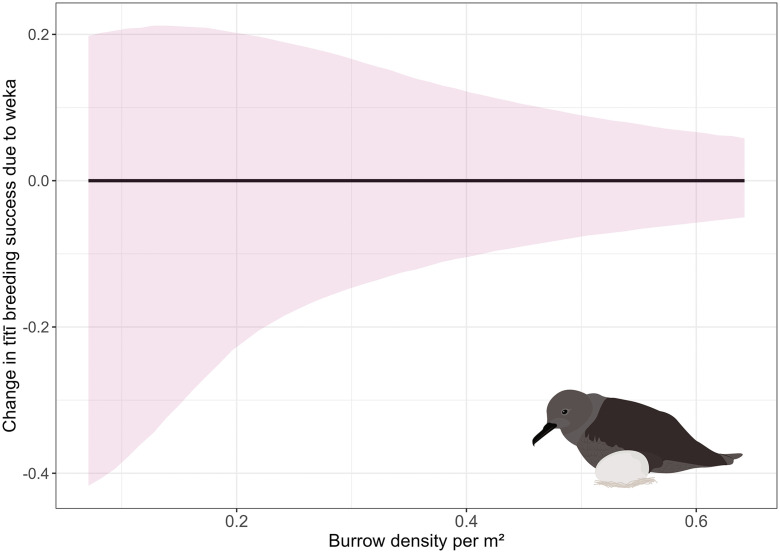
Estimates (black line) and 95% credible intervals (shaded pink) of the change in tītī (*Ardenna grisea)* breeding success due to weka (*Gallirallus australis*). Estimates are derived from model averaging across the models shown in Table 1. The *x*-axis shows the range of observed burrow densities shared across all five study sites.

Tītī breeding success was variable across sites, ranging from 0.39 to 0.88 (Fig 2A), with the WAIC weight for the model involving all four site predictors (Site + Weka) being larger than that for the model involving no predictors (Intercept; Table 1). WAIC weights showed support for models with different combinations of fixed effects for site, weka presence, and burrow density (Table 1), so we used model averaging to obtain parameter estimates. These estimates showed no evidence of an effect of weka presence on tītī breeding success (i.e., the CIs for the predicted impact of weka on breeding success across a range of burrow densities all overlapped zero), but with substantial uncertainty, probably driven by the high variation among sites (Fig 2B; Fig 3). Breeding success increased with increasing burrow density, but again, with considerable uncertainty as shown by the CIs in Fig 2B.

**Table 1:** WAIC (Widely Applicable Information Criterion) model weights for all models. An interaction term is indicated by a colon. For simplicity, we use the following notation: BS = breeding success, BD = burrow density, Weka = the predictor comparing weka and non-weka sites, Site = the three predictors that constitute between-site variation not accounted for by the Weka predictor (see the text for details).

Model	WAIC weight
BS ~ BD + Site + BD:Site	0.58
BS ~ BD + Site + BD:Site + Weka	0.17
BS ~ BD + Site + BD:Site + Weka + BD:Weka	0.10
BS ~ BD + Site	0.09
BS ~ BD + Site + Weka	0.03
BS ~ Site	0.01
BS ~ BD + Site + Weka + BD:Weka	0.01
BS ~ Site + Weka	0.01
BS ~ BD	0.01
BS ~ BD + Weka	<0.01
BS ~ BD + Weka + BD:Weka	<0.01
BS ~ Intercept	<0.01
BS ~ Weka	<0.01

Weka investigated burrows frequently throughout the monitoring period, either by disappearing entirely into the burrow, or by foraging within the entrance. Our trail cameras recorded two different weka individuals removing eggs from two monitored burrows (on 21 Dec 2024 and 1 January 2025), where each weka disappeared into a burrow and then emerged with a dirty egg pierced on its beak, which it then took out of view. We recorded weka preying on chicks at four monitored burrows (three at Parekiore, and one at Manu Maaka), although we also captured evidence of weka preying on chicks from four other unmonitored burrows (one at Pukeweka, and three at Parekiore). Weka usually disappeared down the burrow and then reappeared dragging the chick out of the entrance. Sometimes the chick was still alive as it emerged from the burrow, and was killed by the weka with several strikes of its beak. The earliest observed weka chick predation was on the 17 January 2025, and the latest was the 11 February 2025. All egg and chick predation happened during daylight hours. We also recorded a mioweka removing an egg from a burrow at one of the weka-free sites (Kundy).

We did not observe any other obvious causes of nest failure, except for one potential flooding event where a burrow appeared submerged by water after heavy rain, and two burrows where the egg had been abandoned (both at weka-free sites). Mean burrow lengths ranged from 83–‍113 cm across the five sites. There was no evidence that burrows were longer at the sites with weka: the model predicted that the presence of weka increased burrow length by 20%, but with a 95% credible interval that ranged from a decrease of 39% to an increase of 80%.

### Weka activity

**Fig 4:**
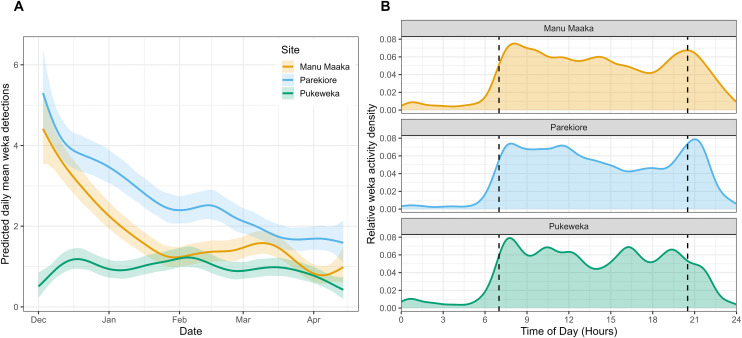
Weka detections and diel activity over the three study sites on the Rakiura Tītī Islands, NZ where weka present. **(A)** Smoothed predictions of mean weka (*Gallirallus australis*) detections (per camera per day) throughout the monitoring period, from a generalised additive model. Means calculated from total weka detections per camera per day (filtering out detections within 5 min of another detection), averaged across each site. Shading shows 95% credible intervals. **(B)** Diel detection patterns of weka during the latter half of the monitoring period (30 January–14 April). Curves show kernel density estimates of independent detection times (5-minute independence threshold), weighted by detection count and scaled so that the total area under each curve equals 1. Thus, the y-axis represents the relative probability distribution of detection time within each site rather than absolute detection rate. Dashed vertical lines indicate mean sunrise and sunset during the sampling period. We only used data from the latter half of the monitoring period as cameras were more consistently active over that time.

The estimate of mean daily weka detections per camera (over all days) was greater at Parekiore (estimate = 2.6, 95% CI = 2.5 to 2.7) than at Manu Maaka (mean = 1.7, 95% CI = 1.6 to 1.8), which was in turn greater than at Pukeweka (estimate = 0.9, 95% CI = 0.9 to 1.0). Parekiore and Manu Maaka showed a peak in weka detections at the start of the monitoring period which then declined, whereas Pukeweka showed a more consistent trend in activity (Fig 4A). There was no indication of an increase in weka activity during the post-guard period when chicks are small and most vulnerable to predation (mid-January until late-February). We also examined the diel activity of weka at the three sites using the camera data from the latter half of the monitoring period between visits 2 and 3 (30 January to 14 April). Weka activity was predominantly diurnal, with small increases at sunrise and sunset (Fig 4B). All three sites showed similar diel patterns.

## Discussion

While the impacts of invasive predators on island faunas have received extensive attention [46,47], dynamics between native predators and their prey in island ecosystems have been less explored. Although weka were introduced to some of the Rakiura Tītī Islands, they co-evolved with tītī across most of NZ, and these islands therefore offer a glimpse into co-evolved predator-prey dynamics in the absence of invasive mammals. Our results demonstrated that sites with weka did not have significantly lower tītī breeding success than sites without weka, although there was considerable uncertainty. Tītī breeding success increased with increasing burrow density.

### Tītī breeding success and the impact of weka

We observed little evidence of a consistent negative impact of weka on tītī breeding success across our limited number of sites, which suggests that at these sites some observed ‘predation’ may actually be scavenging behaviour. For example, the egg predations we observed on camera may have been nests that had already failed and been abandoned, as the eggs were dirty and it seems unlikely that a weka could steal an egg from an incubating adult tītī. Harper [34] also observed weka consuming tītī eggs on Taukihepa, but likewise could not determine whether this was scavenging of abandoned eggs or predation of viable eggs. Some weka predation could also encompass compensatory mortality, where predation replaces mortality that would have occurred even in the absence of weka (e.g., due to starvation). One of our weka sites (Parekiore) did have significantly lower breeding success (probability of an egg becoming a fledgling = 0.39) than the other four sites. However, we cannot necessarily attribute this low breeding success to weka, as the other two weka sites had much higher breeding success (probability > 0.8). Parekiore did have higher daily weka detections than the other two weka sites, which may indicate more abundant weka at this site, and potentially higher predation pressure. Unlike the other two weka sites, there is no hunting of weka at Parekiore, which could have resulted in greater weka abundance there.

Overall, tītī breeding success across all sites was reasonably high (mean of 0.73), although we note that further chick deaths may have occurred after we finished monitoring in mid-April. This breeding success is similar to that found on nearby Codfish Island/Whenua Hou (weka absent) over 9 years’ monitoring (0.76), and double that found on the subantarctic Snares Islands (0.35 over 9 years; weka absent) [36]. Geary et al. [48] also recorded much lower tītī breeding success on a predator-free island in the Marlborough Sounds (0.4). The average breeding success we observed is much higher than that observed for mainland tītī colonies in the presence of invasive mammalian predators, where rates ranged from 0 to 0.48 at four southern South Island sites [49].

We hypothesised 1) that areas with high tītī burrow densities would have higher breeding success, and 2) that this benefit would be enhanced at sites with weka (i.e., the positive slope between breeding success and burrow density would be steeper at sites with weka versus those without weka). While tītī breeding success did improve with increasing burrow density, this effect was similar for sites both with and without weka, probably because we lacked a large enough sample size to properly determine these more subtle relationships. Our results align with previous findings indicating tītī breeding success increases with burrow density up to a threshold (c. 0.6 burrows/m^2^), beyond which it may decline due to negative density-dependence (although this latter prediction is based off a single data point) [42]. Although we observed a similar positive trend up to the 0.6 burrows/m^2^ density threshold, we could not assess what happened beyond this value because our study sites did not have burrow densities > 0.6 burrows/m^2^.

We suggest that, at the island scale, the large, high-density tītī colonies on our study islands likely play a key role in buffering the population against potential impacts of weka predation. Predation in tītī colonies on the NZ mainland by mammalian predators similarly can be inversely density dependent, with the greatest impacts occurring at the smallest colonies [18,20]. Tītī may be a secondary prey item for weka on our study islands, with interannual variation in weka density probably driven by the availability of other primary food sources such as invertebrates and fruit. Although Harper [31] estimated that tītī made up 59% of the weka diet on Taukihepa based on stable isotope analysis of weka muscle tissue, this method reflects only short-term dietary intake (over c. 1–3 months) and coincided with the period when tītī chicks were present. Since tītī chicks are only vulnerable to weka during a brief window (c. 8 weeks from hatching until they are large enough to defend themselves), weka must rely on other resources for most of the year. Additionally, as suggested by Cunninghame [32], tītī predation may be a learned behaviour practiced by a subset of individual weka. If tītī are indeed a secondary, intermittent prey item (and therefore weka populations do not respond numerically to them), then predation by weka may be inversely density dependent: the larger the tītī population at a site, the less significant the impact of weka predation [50]. Further research on drivers of interannual variation of weka density on the Tītī Islands, and their diet outside the tītī breeding season, is needed.

Eradicating invasive mammals from islands can release native and introduced predators from top-down pressure. This release can sometimes cause unintended consequences for native prey populations if the native predator exerts more predatory pressure than the invasive predator [10]. The three sites with weka present all underwent ship rat eradications in the early 2000s, which may have altered interactions between weka and tītī by increasing weka densities [33]. Before eradication, ship rats appeared to be impairing weka breeding success through predation of eggs or chicks [32], and rats would also have competed with weka for fruits and invertebrates. Ship rat eradication is unlikely to have improved tītī breeding success as ship rats were estimated to rarely prey on tītī nests [31], but more study is warranted. Also, the average breeding success we recorded across the two sites on Taukihepa (0.62) was lower than that recorded by Harper [31] in 2003 when ship rats were still present (mean 0.81 across three sites where rats had been briefly suppressed, but weka were present), so weka may be having a greater impact on tītī than they did pre-rat eradication. However, we note that tītī breeding success is naturally variable from year to year primarily due to oceanic conditions [36], so minimal inference can be made from comparing breeding success from 2 years only. Similarly, the effect of weka on tītī that we estimated may change significantly during a year of poor breeding success for tītī, as undernourished chicks may take longer to reach weights where they may be safe from weka predation.

### Weka activity

Our detections of weka on trail cameras provided a coarse index of weka activity throughout the monitoring period. They indicated a decline in activity from early December through the end of January for the two sites on Taukihepa (Parekiore and Manu Maaka), but not the Pukeweka site. It is difficult to speculate what might underlie this pattern, as it could either reflect a decline in weka density, or just a change in their activity. When we arrived at the sites in early December, small weka chicks were present, indicating that weka may be at the height of their breeding cycle and annual abundance (although we note that weka are capable of multiple clutches within a year). Weka chick mortality and juvenile dispersal may have caused a decline in detections.

Interestingly, weka did not show any evidence of increased activity during the time when tītī chicks were small and most vulnerable to predation (i.e., from mid-January until late-February). We did not specifically distinguish our weka detections by whether weka went down the burrow or were just foraging more generally in close proximity, but it appeared that weka continually checked burrows throughout the season. Weka appeared to use burrows to source invertebrate prey like wētā (a flightless orthopteran) which were often observed within the burrows.

Weka were surprisingly diurnal, compared to other studies that have found more crepuscular [51] and nocturnal activity [52]. Despite the nocturnal imaging capabilities of our cameras, all photographed incidents of tītī egg and chick predation occurred during daylight. Tītī dominate presence and ground activity on these islands throughout the night, and have intense periods of activity during late dusk and early dawn as they arrive and depart, respectively. Weka may avoid being active during these times, although one would need data from periods where tītī are absent from the islands to test this.

### Management implications

Tītī populations have undergone varying levels of decline on different Rakiura Tītī islands since the 1960s [21,42]. Population modelling indicated the key driver of decline was lower adult survival and reduced productivity due to unfavourable oceanic conditions associated with El-Niño-Southern Oscillation events, with weka predation, harvesting by humans, and bycatch in fisheries also contributing [21]. As tītī are a long-lived, *k*-selected species, factors that affect adult survival will be the most critical for influencing their population abundance [53,54]. Factors that affect productivity are likely to be less important, but may still influence the ability of tītī populations to recover following the periodic ‘knock-downs’ associated with climate variability and El-Niño events.

We observed a negligible effect of weka on tītī breeding success across our sites, suggesting that management of weka (e.g., through hunting or eradication) may increase productivity only slightly, especially if weka predation is largely a compensatory form of mortality rather than additive. Similarly, recent tītī population modelling predicted that removal of weka predation (using a 20% nest failure rate due to weka at the sites where they are present) would only slightly improve the probability that the tītī population would remain stable or increase in future [55]. However, we note that one of our sites with weka present (Parekiore) did have much lower breeding success, and that we only measured breeding success over a single year. It is also difficult to assess how much weka hunting at Pukeweka and Manu Maaka may have alleviated weka predation pressure on tītī. A further factor to consider is that if tītī populations decline in future this may lead to a higher per capita impact of weka predation.

The subspecies of weka that occurs on the Rakiura Tītī Islands (*Gallirallus australis scotti*) is currently ranked as more threatened than tītī under the NZ Threat Classification System (‘Threatened: Nationally vulnerable’ for Stewart Island weka versus ‘At risk: Declining’ for tītī [56]). It is rare on Rakiura, its previous stronghold, and only thrives on offshore islands around Rakiura where mammals have been eradicated [57]. Decisions regarding potential management of weka on the Rakiura Tītī Islands lie with Rakiura Māori, who are the kaitiaki (environmental guardians) of these islands. The generally accepted reason weka were originally translocated from Rakiura to several of the Rakiura Tītī Islands was to provide an alternative food source for Rakiura Māori muttonbirders during the tītī harvesting season [28]. However, they have also been a valued source of feathers, meat, and oil over the years. As a consequence, different values and perspectives exist within the Rakiura muttonbirding community around whether weka should be removed from the islands or not. These perspectives can vary between muttonbirders from island to island. The management conundrum for Rakiura whānau, therefore, is whether the benefits of having weka on the islands outweighs the potential impact weka have on biodiversity on the islands, including on tītī.

Weka have been eradicated from two Rakiura Tīti Islands thus far (Kundy and Mokonui). An alternative strategy might encompass a more organised, financially supported harvest management approach for weka, that suppresses their densities and potentially reduces predation on tītī and other fauna, whilst still retaining the ecological and cultural values that weka provide. Two of our weka sites already experience some lethal control of weka (Pukeweka and Manu Maaka), and these sites were associated with higher breeding success for tītī. However, more sites would be needed to determine whether this is a causal relationship. Monitoring of outcomes to understand possible cultural and ecological feedbacks associated with a more structured harvest of weka would be critical.

More broadly, our study provides an example of how complex ecological and social trade-offs may begin to emerge as restoration activities progress and the focus shifts from invasive mammals to the ongoing role of predation by native predators. Although we did not find evidence for consistent negative impacts of weka on tītī breeding success, it is important to acknowledge that additional pressures facing native prey species may cause ongoing declines which can then escalate the impact of predation by native predators, even if predator densities and their consumption of prey remain unchanged. Sustainable relationships between native predators and their prey will be a key part of restoring healthy ecosystems, but will need careful adaptive management [58]. Biocultural approaches that explicitly consider cultural and ecological values associated with different species could help provide a path forward [e.g., 29].
